# Analysis of Pathogenic Bacterial and Yeast Biofilms Using the Combination of Synchrotron ATR-FTIR Microspectroscopy and Chemometric Approaches

**DOI:** 10.3390/molecules26133890

**Published:** 2021-06-25

**Authors:** Samuel Cheeseman, Z. L. Shaw, Jitraporn Vongsvivut, Russell J. Crawford, Madeleine F. Dupont, Kylie J. Boyce, Sheeana Gangadoo, Saffron J. Bryant, Gary Bryant, Daniel Cozzolino, James Chapman, Aaron Elbourne, Vi Khanh Truong

**Affiliations:** 1 Nanobiotechnology Laboratory, School of Science, College of Science, Engineering and Health, RMIT University, Melbourne, VIC 3001, Australia; s3741431@student.rmit.edu.au (S.C.); S3601350@student.rmit.edu.au (Z.L.S.); russell.crawford@rmit.edu.au (R.J.C.); sheeana.gangadoo@rmit.edu.au (S.G.); 2 School of Science, College of Science, Engineering and Health, RMIT University, Melbourne, VIC 3001, Australia; madeleine.dupont@rmit.edu.au (M.F.D.); kylie.boyce@rmit.edu.au (K.J.B.); saffron.bryant@rmit.edu.au (S.J.B.); gary.bryant@rmit.edu.au (G.B.); 3 School of Engineering, College of Science, Engineering and Health, RMIT University, Melbourne, VIC 3001, Australia; 4 Infrared Microspectroscopy Beamline, ANSTO Australian Synchrotron, Clayton, VIC 3168, Australia; jitrapov@ansto.gov.au; 5 Centre for Nutrition and Food Sciences, Queensland Alliance for Agriculture and Food Innovation (QAAFI), The University of Queensland, Brisbane, QLD 4072, Australia; d.cozzolino@uq.edu.au

**Keywords:** biofilms, synchrotron, infrared, chemometrics, ATR, spatial heterogeneity

## Abstract

Biofilms are assemblages of microbial cells, extracellular polymeric substances (EPS), and other components extracted from the environment in which they develop. Within biofilms, the spatial distribution of these components can vary. Here we present a fundamental characterization study to show differences between biofilms formed by Gram-positive methicillin-resistant *Staphylococcus aureus* (MRSA), Gram-negative *Pseudomonas aeruginosa*, and the yeast-type *Candida albicans* using synchrotron macro attenuated total reflectance-Fourier transform infrared (ATR-FTIR) microspectroscopy. We were able to characterise the pathogenic biofilms’ heterogeneous distribution, which is challenging to do using traditional techniques. Multivariate analyses revealed that the polysaccharides area (1200–950 cm^−1^) accounted for the most significant variance between biofilm samples, and other spectral regions corresponding to amides, lipids, and polysaccharides all contributed to sample variation. In general, this study will advance our understanding of microbial biofilms and serve as a model for future research on how to use synchrotron source ATR-FTIR microspectroscopy to analyse their variations and spatial arrangements.

## 1. Introduction

Numerous bacteria and fungi form biofilms to aid in their reproduction and proliferation. Biofilms are assemblages of microbial cells embedded within a matrix of extracellular polymeric substances (EPS) (such as polysaccharides, DNA, proteins, and lipids) and components obtained from the surrounding environment [1,2]. These biofilms can be found on a variety of biotic and abiotic substrate surfaces [3]. Microbial cells behave differently in biofilms than they do in the planktonic state, resulting in altered gene expression and cell proliferation [4]. Cells that form biofilms possess survival strategies tailored to the particular microbial species involved, such as increased virulence [3] and protection from antimicrobial agents [5,6], harsh environmental conditions [3], and the human immune system [7]. Quorum sensing is used by the microbial cells inside the biofilm to facilitate cell-to-cell communication and horizontal gene transfer, which increases the development of desirable traits, such as drug tolerance [8]. The proliferation of biofilms on surfaces is an issue known as biofouling. Biofouling manifests as enhanced rates of corrosion of metal structures [9], contamination of water systems [10,11], and increased risk of infection in food production [12,13] and healthcare [14], among other settings [9]. Notably, the accumulation of pathogenic biofilms on implanted medical devices results in substantial morbidity, death, and financial costs for both patients and healthcare systems [13,14]. Microbial biofilms present a greater treatment challenge than planktonic bacteria, necessitating the implementation of novel treatment strategies [15,16].

The biochemical composition of the biofilm matrix can vary significantly between different strains and growth conditions [17,18]. The spatial heterogeneity of microbial cells and other biofilm components has been identified as a critical property for biofilm function, resulting in microdomains with variable biochemical composition [19,20]. Being able to map these microdomains can lead to a greater understanding of the complex processes occurring within the biofilm [19]. Different techniques have been employed to map the biochemical spatial arrangement of biofilm components [9,21,22,23,24,25]. Some studies have focused on Fourier-transform infrared (FTIR) imaging [26,27,28], or near-infrared spectroscopy (NIR), which utilizes vibrational spectroscopy to exploit the absorption of light in the near-IR region, as the molecular resonant frequency varies with differences in electronegativity, size, orientations, and/or the molecular environments of the specific molecular species [29]. This technique allows the detection of certain functional groups which are indicative of specific chemical species. The main components of microbial biofilms are lipids, proteins, and polysaccharides, many of which have well-defined vibrational modes in the infrared range of the electromagnetic spectrum [30]. FTIR microspectroscopy can be used for the spatial mapping of samples through spectra acquisition at many points across the sample [26,31], while traditional benchtop FTIR instruments can get to resolutions of a few microns [32].

Additionally, the use of highly collimated synchrotron light for FTIR analysis enables sub-micron spatial mapping and can achieve higher signal-to-noise ratios, allowing for the identification of less prominent peaks in the spectrum associated with specific chemical species [29,33]. Synchrotron-sourced FTIR has been utilized to investigate the biological samples [26,29,33]. To our knowledge, this is the first research to analyze bacterial biofilms using synchrotron macro attenuated total reflectance (ATR)-FTIR microspectroscopic technique. 

The chemical properties of monoculture biofilms produced by methicillin-resistant *Staphylococcus aureus* (MRSA), *Pseudomonas aeruginosa*, and *Candida albicans* are investigated in this work. These pathogens were selected as representative of Gram-positive and Gram-negative cells, and yeast, respectively. They are all human pathogens that infect at a high rate through biofilms [34,35,36]. Synchrotron macro ATR-FTIR microspectroscopy at the Australian Synchrotron was utilized to obtain high-resolution spectral maps of the three pathogenic microbial biofilms. These maps were fully analyzed using chemometric approaches such as multivariate statistical methods. This study adds to our understanding of microbial biofilms and demonstrates the utility of synchrotron macro ATR-FTIR microspectroscopy for biofilm analysis.

## 2. Results and Discussion

### 2.1. Biofilm Morphological Characterisation

**Figure 1:**
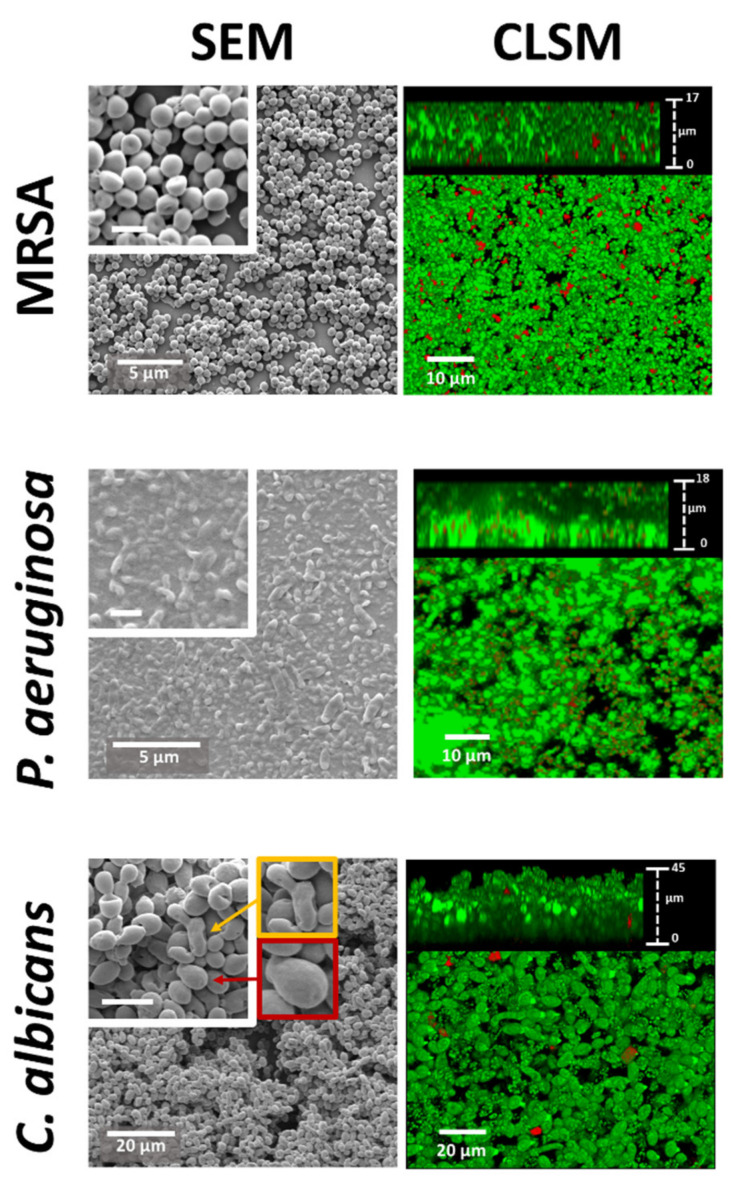
SEM and CLSM images showing the morphology of pathogenic biofilms formed by MRSA, *P. aeruginosa* and *C. albicans*. An example of an early pseudohyphal cell (yellow) and yeast cell (red) are highlighted in the *C. albicans* biofilm. Scale bars in the insets of the electron micrographs are 1 µm for the two bacterial species and 5 µm for *C. albicans*.

MRSA, *P. aeruginosa* and *C. albicans* were used to prepare biofilms on glass surfaces. These pathogens specifically cause a significant number of biofilm-associated infections on biomedical devices [34,35,36]. The biofilms were visualised using scanning electron microscopy (SEM) and confocal laser scanning microscopy (CLSM) imaging (Figure 1). The SEM images show that all three pathogenic microorganisms formed thick biofilms. Excess non-cellular biomass, presumably composed of EPS, was observed binding individual cells in the *P. aeruginosa* biofilm, while this non-cellular biomass was less prevalent in the MRSA biofilm. The biofilm of *C. albicans* was dominated by yeast cells, but it also contained some elongated cells that may have been emerging pseudohyphal cells. Different cell morphologies within *C. albicans* biofilms have been previously examined [37,38]. This biofilm morphology represents an early-intermediate phase of biofilm formation for *C. albicans* before significant EPS production [39,40].

CLSM can be used to generate three-dimensional images and data of the biofilms (Figure 1). The biofilms were stained with fluorescent dyes, which allowed for the visualisation of cells with intact membranes (coloured green) and cells with membrane damage (coloured red). These biofilms were thick (>15 µm in all cases) and mostly consisted of green cells with intact membranes, with only a few red cells, suggesting an overall stable biofilm.

### 2.2. Analysis of Microbial Biofilms Using Synchrotron Macro ATR-FTIR Microspectroscopy

The microbial biofilms were analysed using macro ATR–FTIR microspectroscopy, generating high-resolution spectral-spatial maps. The spectral region ~3900–750 cm^−1^ was examined, which includes spectral regions where chemical species indicate the significant components of microbial biofilms, e.g., proteins, lipids and polysaccharides absorb light [30,41]. Table S1 lists the major lipids, proteins, and polysaccharides found in each biofilm used in this analysis. Prior to the analysis, the spectra were smoothed, baseline corrected, and normalized using the OPUS 8.0 program. Table 1 and Table S2 show the functional groups that were assigned to IR spectral regions.

**Figure 2:**
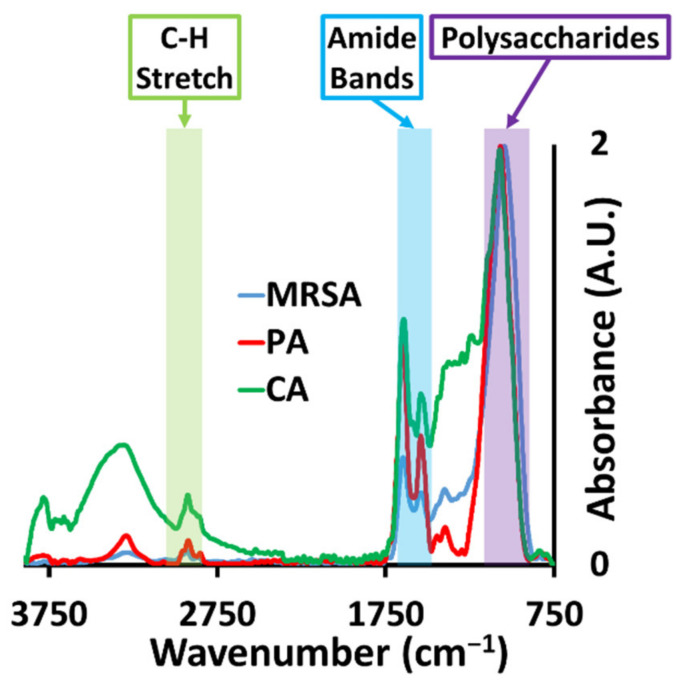
Average macro ATR-FTIR spectra for the three biofilms. MRSA, PA (*P. aeruginosa*) and CA (*C. albicans*). Regions characteristic of chemical species of interest are highlighted.

The average macro ATR-FTIR spectra for the three biofilms are presented in Figure 2. These averages were derived from 1600 individual spectra for each of the bacterial species and 4200 spectra for *C. albicans*, reflective of the larger size of the yeast pathogen. The spectral regions containing characteristic absorption peaks of major chemical species are highlighted. All three biofilms exhibit strong absorptions in the regions assignable for lipids (3000–2800 cm^−1^), proteins (1705–1600 cm^−1^) and polysaccharides (1200–950 cm^−1^). The averaged sample spectra are distinct from each other. To the author’s knowledge, this is the first study to demonstrate the use of synchrotron sourced FTIR as a discriminating technique between microbial biofilms of different species. 

**Figure 3:**
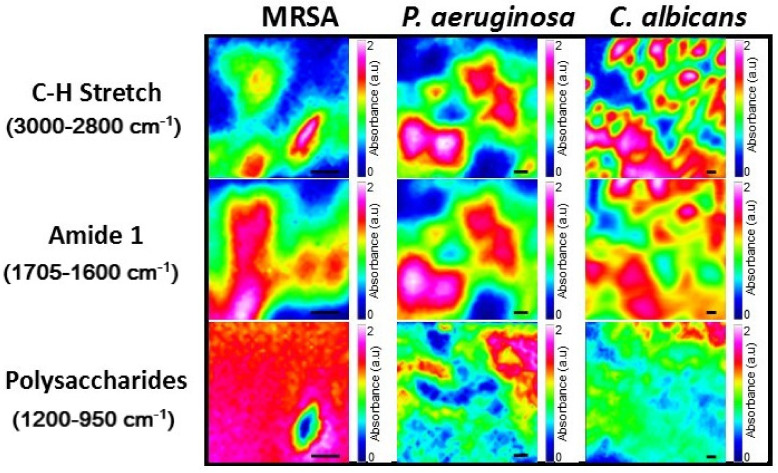
Synchrotron macro ATR-FTIR spectral maps of the biofilms formed by MRSA, *P. aeruginosa* and *C. albicans*. All scale bars are 2 µm.

Spectral maps of the microbial biofilms were generated by integrating the area under the absorption peaks within the specified regions (Figure 3 and Figure S1). The spectral maps display spatial variation within the biofilms for all spectral regions and microbial species, demonstrating the chemical heterogeneity exhibited within biofilms [2]. Interestingly, the C-H stretch and amide I regions were homologous primarily to each other, for each microbial species respectively. This relationship may be explained by the localization of high concentrations of cellular materials in those regions, which possess biomolecules containing these chemical groups and potentially represent a clustering of cells.

### 2.3. Comparison of Amide I-Rich Regions of Biofilms through Multivariate Data Analysis

**Figure 4:**
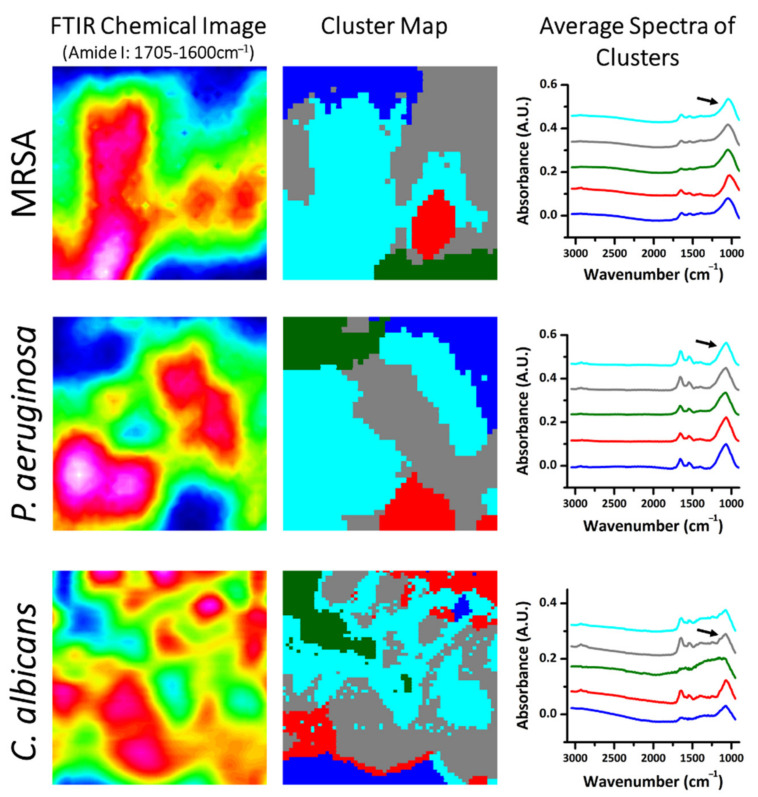
HCA maps of biofilm samples to identify and group regions high in amide I. The FTIR chemical image of the Amide I band (left), the HCA map of the 5 clusters (middle) and the average spectra of the clusters (right). The black arrows refer to the average spectrum, representative of the same colored cluster, which was chosen for further PCA analysis.

The study of multivariate data revealed differences between microbial biofilm samples. Multivariate data analysis is useful for revealing information in complex data sets that would otherwise be missed by simpler analysis, particularly in natural systems, which are inherently multi-faceted [44,45]. The spectral data were pre-processed by cutting out the biological regions of interest, namely 1755–910 cm^−1^ and 3035–2780 cm^−1^, which were used for analysis. Cluster maps are presented in Figure 4 which show groupings of areas with different spectra. These maps were generated from hierarchical cluster analysis (HCA) using the 2nd derivative of the spectral data. The maps show distinct clusters of several micrometers in diameter. The HCA functioned as a quality control test, with HCA clusters chosen for further analysis based on two criteria: (1) low signal-to-noise ratio, and (2) prominent peaks in the amide I region (1705–1600 cm^−1^). These criteria were chosen as markers for cell-rich regions and represent good quality spectra, useful for further multivariate analysis.

**Figure 5:**
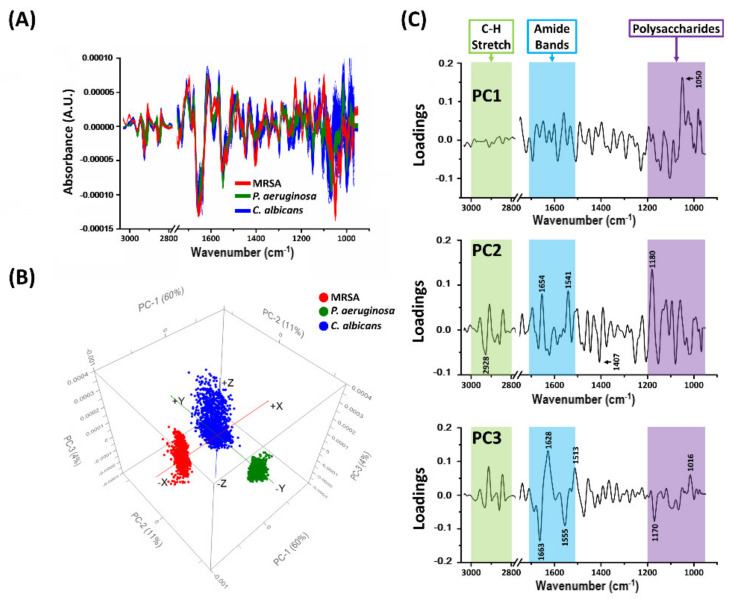
PCA analysis of the 2nd derivative spectra for each biofilm sample. (**A**) Second derivative spectra for each biofilm sample. (**B**) PCA score plots showing projections against the first 3 PCs that explain the majority of the spectral variation. (**C**) PC loading plots for the first 3 PCs. The wavenumbers at which significant change occurs are designated.

The 2nd derivative of each spectrum within the cluster of interest for each biofilm (detailed in Figure 4) was processed using extended multiplicative scatter correction (EMSC). The results of the princiapl component analysis (PCA) are presented in Figure 5. The 2nd derivative spectra show dominant peaks (maxima) and troughs (minima) in the amide region for all three biofilms (1600–1705 cm^−1^; Figure 5A and Figure S2). The polysaccharides region, 950–1200 cm^−1^, also showed differences between the different species biofilm. The PCA score plot shows a clear separation between the three microbial samples (Figure 5B). Most of the variance between the three samples can be explained by the PC1 and PC2, while the PC3 separates individual spectra within each microbial sample. 

The PC loading plots are presented in Figure 5C. The loading plot of PC1 shows a notable peak in the polysaccharide region (1050 cm^−1^). This peak is likely to refer to ν(C–O) coupled with δ(C–O) of C–OH groups of carbohydrates [46,47,48], and is the major contributor towards the observed differences between species in the PCA score plot (Figure 5B). The wavenumbers where notable peaks and troughs occur in the PCA loading plots were assigned with their likely functional group, derived from a literature search, and are presented in Table S3.

When these findings are combined, it is clear that there are discernible variations in the FTIR spectra of the biofilms produced by the three pathogenic microorganisms examined. These distinctions are due to the structural and chemical composition of the three biofilms. The variations between microbial biofilms can be better understood by using multivariate data analysis, which allows for a better understanding of the different biomolecular functional groups presented in various wavelengths that contribute to the differences between and within the different biofilms. The spectral range of polysaccharides was the most important contributor to the variations between the biofilm samples. These findings support the use of synchrotron macro ATR-FTIR microspectroscopy as a novel analysis technique for studying microbial biofilms, by enabling the chemical mapping measurements to be performed at sub-micron scale.

## 3. Materials and Methods

### 3.1. Strains, Growth Conditions, and Sample Preparation

Biofilms were grown from two strains of pathogenic bacteria, *Staphylococcus aureus* ATCC BAA-1680, *Pseudomonas aeruginosa* ATCC 27853, which were obtained from the American Type Culture Collection (ATCC, Manassas, VA, USA), and one strain of pathogenic yeast, *Candida albicans* clinical isolate 18-29511395, sourced from South Australia Pathology—Cellular Therapies Laboratory. These species were selected as representative of a Gram-positive and Gram-negative bacteria, and a representative yeast, respectively, all of which cause a significant number of biofilm-associated infections on biomedical devices [34,35,36]. Before each experiment, bacterial cultures were refreshed on lysogeny broth (LB) agar (B.D., U.S.A.) from stocks and grown for 24 h at 37 °C. Yeast cultures were refreshed on potato dextrose broth (PDB) agar at 25 °C for 48 h. Bacterial and yeast suspensions were adjusted to OD600 = 0.3. A total of 2 ml of microbial solution was used for all biofilm experiments. Biofilms were grown on individual glass-bottom cell culture dishes (World Precision Instruments, Sarasota, FL, USA) for CLSM and SEM experiments, and pathology grade glass slide substrates (Livingstone, Australia) for ATR-FTIR analysis at static conditions for 24 h at 37 °C. The glass slide substrates were immersed in the microbial solution to prevent the possibility of their drying out.

### 3.2. Scanning Electron Microscopy (SEM) Characterisation

Electron micrographs were obtained using a FEI Verios 460L SEM (FEI Company, Hillsboro, OR, USA), measuring secondary electrons and operated with an accelerating voltage of 2–5 kV. For cellular imaging, all samples were affixed using formaldehyde/glutaraldehyde 3% solution in 0.1 M sodium cacodylate buffer (pH~7.4) (ProSciTech, Thuringowa Central, Australia) rinsed, dehydrated using a series of 30%, 50%, 75%, 90%, and 100% ethanol solutions, and coated with a thin film of conductive gold or iridium before imaging.

### 3.3. Confocal Laser Scanning Microscopy (CLSM) Characterisation

Confocal laser scanning microscopy (CLSM) was performed on an Olympus Fluoview FV1200 inverted microscope and a ZEISS LSM 880 Airyscan upright microscope using a 63× immersion lens. Biofilms were washed gently three times with PBS to remove any planktonic or poorly attached cells. Cells were dyed using a LIVE/DEAD BacLight Bacterial Viability Kit (including SYTO 9 and propidium iodide). Bacterial biofilms were stained according to the manufacturer’s protocol [49]. Z-stack imaging was utilized to generate reconstructed three-dimensional images of the biofilms using the Zen Black software (Zeiss, Oberkochen, Germany).

### 3.4. Synchrotron Macro Attenuated Total Reflectance-Fourier Transform Infrared (ATR-FTIR) Microspectroscopy

Synchrotron macro ATR-FTIR microspectroscopic analysis of the microbial biofilms was performed at the Infrared Microspectroscopy (IRM) beamline at the Australian Synchrotron using a Bruker Hyperion 2000 FTIR microscope equipped with a liquid nitrogen-cooled narrow-band mercury cadmium telluride (MCT) detector, coupled to a VERTEX V80v FTIR spectrometer (Bruker Optik GmbH, Ettlingen, Germany).

The spatially resolved distribution of the chemical functional groups present in the MRSA, *P. aeruginosa*, and *C. albicans* biofilms was imaged in macro attenuated total reflection–Fourier transform infrared (ATR–FTIR) mapping mode, as previously described in [29]. An in-house developed macro ATR–FTIR device equipped with a 250 μm diameter facet germanium (Ge) ATR crystal (nGe = 4.0) and a 20× IR objective (NA = 0.60; Bruker Optik GmbH, Ettlingen, Germany) was used. The unique combination of the high refractive index of the Ge ATR crystal and the high NA objective used in this device, when coupled to the synchrotron-IR beam, allowed the surface characterization of the microbial biofilm samples to be performed at a high spatial resolution < 1 µm.

The biofilms were grown on pathology grade glass slide substrates (Livingstone, Australia) and were fixed with formaldehyde/glutaraldehyde 3% solution in 0.1 M sodium cacodylate buffer (pH~7.4) (ProSciTech, QLD, Australia) to preserve their structure, air-dried, and mounted on an aluminum disc. The aluminium disc was then placed into the sample stage of the macro ATR–FTIR unit. Subsequently, the Ge ATR crystal was brought to the focus of the synchrotron-IR beam, and a background spectrum was recorded in air using 4 cm^−1^ spectral resolution and 256 co-added scans. The microbial biofilm samples were then brought into contact with the sensing facet of the Ge ATR crystal, and a synchrotron macro ATR–FTIR chemical map was acquired. The penetration depth of this technique is <2 µm, resulting in spectra measured from the top of the biofilm. Every spectrum was collected with a beam defining aperture providing a nominal measurement area of 3.13 μm diameter per pixel, at 0.5 μm step intervals. For each pixel, the synchrotron macro ATR–FTIR spectrum was recorded within a spectral range of 3900–750 cm^−1^ using 4 cm^−1^ spectral resolution and 8 co-added scans. Blackman-Harris 3-Term apodisation, power-spectrum phase correction, and a zero-filling factor of 2 were set as the default acquisition parameters using the OPUS 8.0 software suite (Bruker), which was also used for the initial data analysis. Prior to analysis, the spectra were smoothed (25 points), the baseline was corrected using a concave rubberband correction (10 iterations and 64 baseline points), and the spectra were normalized using vector normalization. Chemical maps were generated from the embedded spectra by integrating the area under the relevant peaks using the OPUS 8.0 software.

### 3.5. Hierarchical Cluster Analysis (HCA) and Principal Component Analysis (PCA)

The multivariate data analysis including HCA and PCA was performed using CytoSpec v. 1.4.02 (Cytospec Inc., Boston, MA, USA) and the Unscrambler X 10.1 software package (CAMO Sofware AS, Oslo, Norway), respectively. The HCA functioned as a quality control test, with HCA clusters chosen for further analysis based on two criteria: (1) low signal-to-noise ratio, and (2) prominent peaks in the amide I region (1705–1600 cm^−1^). Spectral wavenumbers covering 1755–910 cm^−1^ and 3035–2780 cm^−1^ were chosen for analysis, as these regions contain the molecular information most relevant to the microbial biofilm samples [41]: in particular, the protein, lipid, and carbohydrate signals (as summarized in Table 1). HCA analysis was carried out with Ward’s algorithm, and cluster imaging was carried out with the processed 2nd derivative spectra by assigning five clusters to be generated.

Prior to PCA, 2nd derivative spectra were similarly generated using the 9 smoothing data points and a polynomial order of 3, by a Savitzky–Golay algorithm which removed the broad baseline offset and curvature [50]. In addition, the 2nd derivative spectra were further processed by extended multiplicative scatter correction (EMSC). In essence, the EMSC algorithm removes light-scattering artefacts and normalizes the spectra to account for pathlength differences [51]. This has been used previously for the FTIR analysis of microbial biofilms [52]. After EMSC, the PCA was performed using the Unscrambler X 10.1 software package.

## 4. Conclusions

In this study, three pathogenic microbial biofilms were analyzed using synchrotron macro ATR-FTIR microspectroscopy. The presence of the major groups of biomolecules was determined for each biofilm. Multivariate analysis was able to separate the three biofilms based on their spectra. The likely chemical functional groups contributing to the significant differences between and within each sample were also presented. This work contributes to a greater understanding of microbial biofilms and presents the use of synchrotron ATR-FTIR microspectroscopy as a useful analysis technique for microbial biofilms.

## Figures and Tables

**Table 1:** Functional groups and their designation to IR spectral regions as used in this study [29,30,42,43].

Functional Group	Spectral Region (cm^−1^)
Fatty acids and lipids (C-H stretching vibrations—inclusive of CH_2_ and CH_3_ functional groups) *	3000–2800
Amide I (C=O stretching of amides)	1705–1600
Polysaccharides (vibrations of C–OH, C–O–C, C–C and PO_2_ stretching)	1200–950

* C-H vibrations occur in additional biomolecules.

## Data Availability

Data is available upon reasonable request.

## Supplementary Materials

The following are available online, Figure S1: Further spectral maps of the pathogenic biofilms, Figure S2: Average 2nd derivative spectra for each biofilm sample, Figure S3: Cross-validation of synchrotron-sourced IR spectra, Table S1: Relevant biomolecular components of the microbial biofilms, Table S2: Functional groups and their designation to IR spectral regions, Table S3: FTIR band assignments for functional groups found in the PCA loadings. [53,54,55,56,57,58,59,60,61,62,63,64,65,66,67,68] is cited in Supplementary Materials.
