# Supplementary material for: Analysis of Pathogenic Bacterial and Yeast Biofilms Using the Combination of Synchrotron ATR-FTIR Microspectroscopy and Chemometric Approaches
Source: Molecules. 2021 Jun 25;26(13):3890. doi: 10.3390/molecules26133890 (PMC8271424; doi:10.3390/molecules26133890)
Supplement: Supplementary file 1 [file molecules-26-03890-s001.zip › molecules-1253231-supplementary.pdf]

Analysis of pathogenic bacterial and yeast biofilms using the combination of synchrotron ATR-FTIR microspectroscopy and chemometric approaches

Samuel Cheeseman[a, b#], Z. L. Shaw[a, c#], Jitraporn Vongsvivut[d], Russell J. Crawford[a,b], Madeleine F. Dupont[b], Kylie J. Boyce[b], Sheeana Gangadoo[a,b], Saffron J. Bryant[b], Gary Bryant[b], Daniel Cozzolino[e*], James Chapman[a,b*], Aaron Elbourne[a,b*] and Vi Khanh Truong[a,b*]

[a]Nanobiotechnology Laboratory, School of Science, College of Science, Engineering and Health, RMIT University, Melbourne VIC 3001, Australia.

[b]School of Science, College of Science, Engineering and Health, RMIT University, Melbourne VIC 3001, Australia

[c]School of Engineering, College of Science, Engineering and Health, RMIT University, Melbourne VIC 3001, Australia

[d]Infrared Microspectroscopy Beamline, ANSTO Australian Synchrotron, Clayton, Victoria 3168, Australia

[e]Centre for Nutrition and Food Sciences, Queensland Alliance for Agriculture and Food Innovation (QAAFI), The University of Queensland, Brisbane

*Corresponding authors

#S.C. and Z.L.S. contributed equally to this work

For correspondence. (V.K. Truong) Email: vi.khanh.truong@rmit.edu.au

Supporting Information:

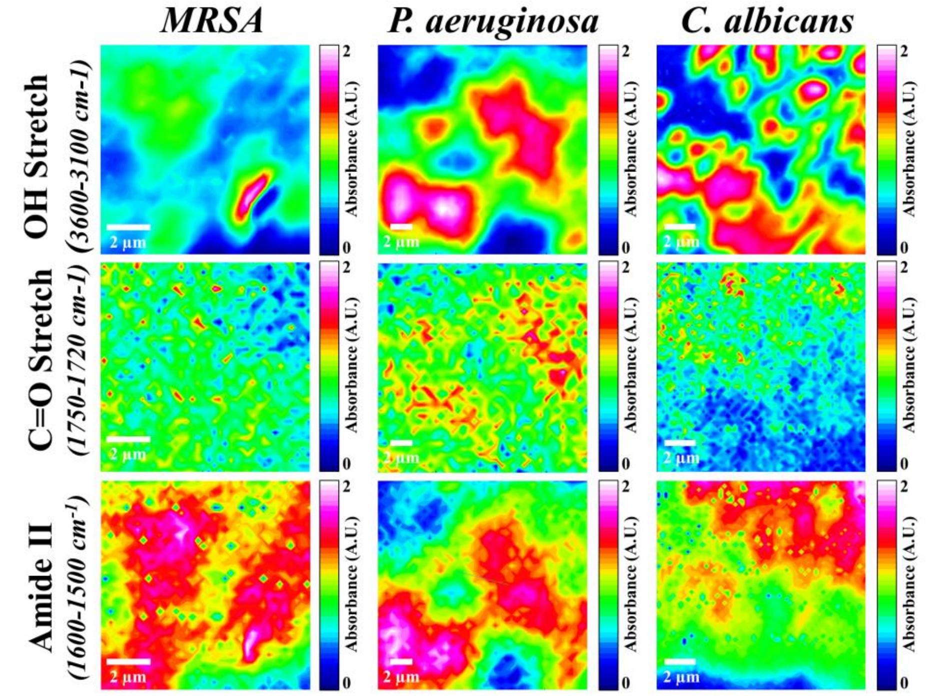

Figure S1. Spectral maps integrated at the OH stretch (3600-3100 cm^{-1}), C=O stretch (1750-1720 cm^{-1}) and amide II bands (1600-1500 cm^{-1}) of the pathogenic biofilms formed by MRSA, *P. aeruginosa* and *C. albicans*.

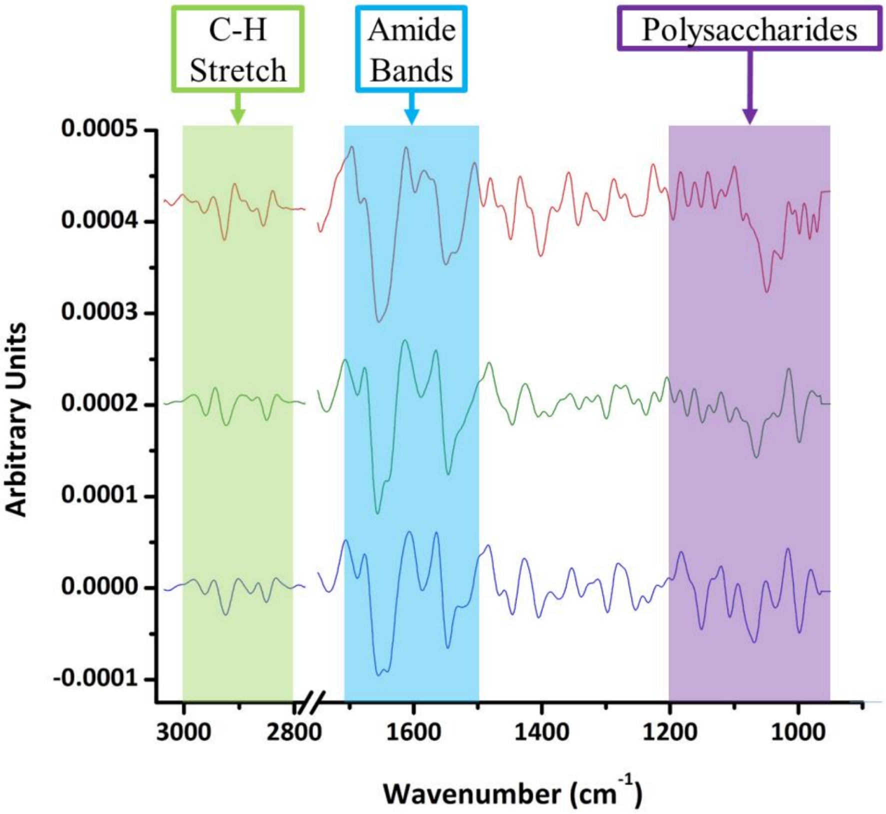

Figure S2. Average 2nd derivative spectra for each biofilm sample.

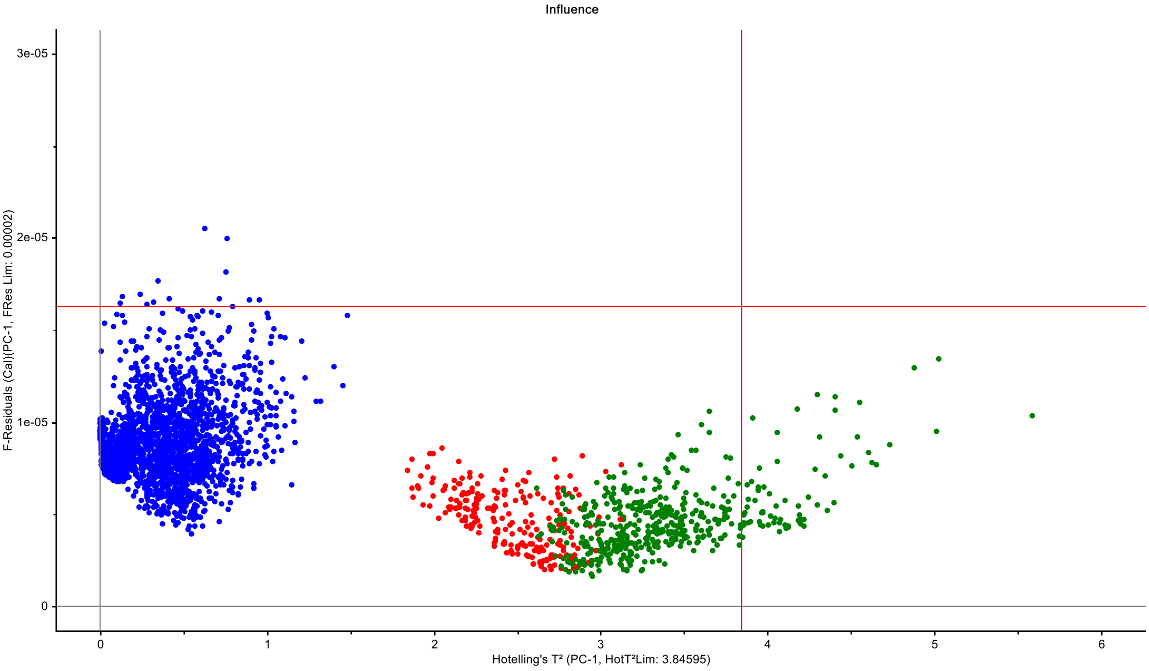

Figure S3. Cross-validation of synchrotron-sourced IR spectra (blue: *Candida albicans;* red: methicillin-resistant *Staphylococcus aureus;* and green: *Pseudomonas aeruginosa*).

Table S1 Relevant biomolecular components (from the literature) of the microbial biofilms analysed in this study

	**Lipids**	**Proteins**	**Polysaccharides**
**MRSA** [53-57]	• Phosphatidylglycerol (PG)<br>• Lysyl-phosphatidylglycerol (LPG)<br>• Cardiolipin (CL)	• *S. aureus* protein A (Spa)<br>• Fibrinogen-binding proteins (FnBPA and FnBPB)<br>• *S. aureus* surface protein (SasG)<br>• Biofilm-associated protein (Bap) and Bap-like proteins<br>• Clumping factor A (ClfA) and B (ClfB)<br>• Extracellular adherence protein (Eap)<br>• Beta toxin (Hlb)<br>• Amyloid fibers<br>• SarA<br>• Penicillin-binding protein 2A (PBP2A)	• Polysaccharide intercellular adhesin (PIA)<br>• Polymeric *N*acetyl-glucosamine (PNAG)
P. aeruginosa [58-61]	• Phosphatidylethanolamine (PE)<br>• Phosphatidylglycerol (PG)<br>• Cardiolipin (CL)<br>• Lipopolysaccharide (LPS) component – lipid A<br>• Rhamnolipids	• Tyrosine phosphatase (TpbA)<br>• CdrA<br>• Large adhesive proteins (Lap)<br>• Lectins (LecA and LecB)<br>• Flagella (flagellin)<br>• Type IV pili (pillins)<br>• TasA amyloid protein	• Alginate<br>• Pel<br>• Psl
C. albicans [38,62-65]	• Phosphatidylcholine (PC)<br>• Lyso-phosphatidylcholine (lyso-PC)<br>• Phosphatidylethanolamine (PE)<br>• Lyso-phosphatidylethanolamine (lyso-PE)<br>• Phosphatidylinositol (PI)<br>• Phosphatidylserine (PS)<br>• Phosphatidic acid (PA)<br>• Glycerolipids<br>• Sphingolipids<br>• Ergosterol<br>• Prostaglandin E2	• Agglutinin-like sequence (Als) proteins<br>• Hyphal wall protein 1 (Hwp1)<br>• Grp2p<br>• Regulatory proteins (transcription factors)<br>• Enzymes (secreted and non-secreted i.e. from lysed cells)	• Mannans<br>• Glucans

Table S2 Functional groups and their designation to IR spectral regions as used in this study [29,30,42,43]

**Functional Group**	**Spectral Region**
Hydroxyl (O-H stretching)	3600-3100
carboxylic acids and ester functional groups (C=O stretching)	1750-1720
Amide II (N-H bending)	1600-1500

Table S3. FTIR band assignments for functional groups found in the PCA loadings

**Wavenumber (Peak)**	**Assignment of Chemical Functional Group**[a]
**PC1**	
1050	ν(C–O) coupled with δ(C–O) of C–OH groups of carbohydrates [46-48]
**PC2**	
2928	ν_{as}(C–H) from methylene (–CH_2) groups of lipids [47,48,66]
1654	Amide I: α–helix [48,47]
1541	Amide II: perpendicular modes of the α–helix and antiparallel β–sheet [47,48,67,68]
1407	ν_s(C=O) of COO- groups [47,66]
1180	ν_s(C–O–C) from esters [47,48]
**PC3**	
1663	Amide I: β–turn [48]
1628	Aggregated β–sheet structures [47]
1555	Amide II band NH bending vibration and CN stretch [66]
1513	Amide II: parallel mode of the α–helix [48]
1170	ν_s(C-O) of hydrogen bonded groups [46]
1016	ν(C–C)$_{skeletal}$ coupled with δ(CH_2) of α–CH_2 in –CH_2OH groups of polysaccharides [48]

[a]ν_{as} = asymmetric stretch; ν_s = symmetric stretch; δ = in-plane deformation (bend).
